# Effect of hydrogen diffusion in an In–Ga–Zn–O thin film transistor with an aluminum oxide gate insulator on its electrical properties†

**DOI:** 10.1039/c7ra12841j

**Published:** 2018-02-01

**Authors:** Yunyong Nam, Hee-Ok Kim, Sung Haeng Cho, Sang-Hee Ko Park

**Affiliations:** Smart & Soft Materials & Devices Laboratory (SSMD), Department of Materials Science and Engineering, Korea Advanced Institute of Science and Technology (KAIST) 291 Daehak-ro, Yuseong-gu Daejeon 34141 Korea shkp@kaist.ac.kr; ICT Materials & Components Research Laboratory, Electronics and Telecommunications Research Institute (ETRI) 218 Gajeong-ro, Yuseong-gu Daejeon 34129 Korea

## Abstract

We fabricated amorphous InGaZnO thin film transistors (a-IGZO TFTs) with aluminum oxide (Al_2_O_3_) as a gate insulator grown through atomic layer deposition (ALD) method at different deposition temperatures (*T*_dep_). The Al_2_O_3_ gate insulator with a low *T*_dep_ exhibited a high amount of hydrogen in the film, and the relationship between the hydrogen content and the electrical properties of the TFTs was investigated. The device with the Al_2_O_3_ gate insulator having a high H content showed much better transfer parameters and reliabilities than the low H sample. This is attributed to the defect passivation effect of H in the active layer, which is diffused from the Al_2_O_3_ layer. In addition, according to the post-annealing temperature (*T*_post-ann_), a-IGZO TFTs exhibited two unique changes of properties; the degradation in low *T*_post-ann_ and the enhancement in high *T*_post-ann_, as explained in terms of H diffusion from the gate insulator to an active layer.

## Abstract 2

We fabricated amorphous InGaZnO thin film transistors (a-IGZO TFTs) with aluminum oxide (Al_2_O_3_) as a gate insulator grown through atomic layer deposition (ALD) method at different deposition temperatures (*T*_dep_).

## Graphical abstract

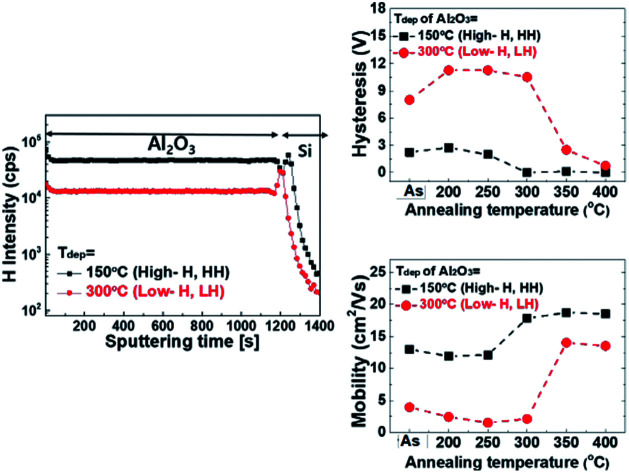

## 1. Introduction

Amorphous oxide semiconductors (AOSs) have been studied intensively as active channel layers for thin film transistors (TFTs) for next-generation displays owing to their scalability and high mobility. Generally, Zn-based AOSs are n-type semiconductors with mobility levels proportional to the carrier concentration.^1^ Among the various AOSs, In–Ga–Zn–O (a-IGZO), which is the best known composition, can exhibit a wide range of electron densities (*N*_e_) ranging from 10^11^ to 10^19^ cm^−3^.^2^ Although a higher *N*_e_ is preferred for high mobility, this value must be carefully controlled because an a-IGZO TFT for which *N*_e_ is too high cannot be turn-off. In a-IGZO films, various intrinsic defects such as metal/oxygen vacancies and interstitials exist, and they mainly determine the electrical properties of the oxide TFT. Many studies have been conducted in an effort to reveal the effects of such defects on a-IGZO TFTs, and it is accepted that oxygen vacancies (V_O_) serve as shallow donors in a-IGZO film and as a source of free carriers, while weakly bonded O acts as an electron trapping center.^1,2^ Therefore, controlling O in a-IGZO film is a key factor to optimize the electrical property of the TFT.

In addition to such intrinsic defects, hydrogen, as an impurity, can also affect the electrical properties of a-IGZO TFTs. Generally, it is known that hydrogen in crystalline-oxide semiconductors (*e.g.*, c-ZnO and c-In_2_O_3_) acts as a source of high conductivity.^3–6^ Hydrogen in ZnO can exist in the form of interstitial H (H_i_) bonded with an oxygen atom and substitutional H (H_O_) located at an oxygen site. In both cases, positive charge states (H_i_^+^ and H_O_^+^) are stable and act as a shallow donor in ZnO. Likewise, in a-IGZO, the role of hydrogen is mainly understood as a shallow donor, generating free carriers. It was revealed that the a-IGZO film itself has a high-density of hydrogen of 10^20^ to 10^21^ cm^−3^.^7^ Because excess H in an a-IGZO layer can lead to difficulty related to the control of *V*_on_ in the TFT, unexpected H should be avoided.

Recently, in several in-depth studies of H in oxide TFT, various effects were reported. Nomura *et al.* reported that all of H do not increase the conductivity of a-IGZO film due to the compensation of the free electrons by excess oxygen.^7^ Furthermore, an interesting role of H was reported in terms of defect passivation. Tsao *et al.* and Hanyu *et al.* reported an improvement in the transfer characteristics of a-IGZO TFTs when H was incorporated during active layer deposition and a post-annealing process, respectively.^8,9^ In addition, a beneficial effect of hydrogen on the reliability of a-IGZO TFTs was reported.^10,11^ However, the opposite effects of hydrogen, where it generates defect states and induces instability during photo-bias stress, were also reported.^12–15^

As described thus far, the role of H in a-IGZO TFTs remains unsolved. In addition, the difficulty in precise control of H in a-IGZO TFT makes the problem more difficult. Because H can be easily incorporated and/or diffused into the a-IGZO layer during the fabrication of TFT devices, an experimental design capable of revealing the role of H in a-IGZO TFT is not straightforward. Specifically, SiO_2_ and SiN_*x*_ layers, which are widely used as gate insulators or passivation layers, are deposited through a plasma-enhanced chemical vapor deposition (PE-CVD) process, which can induce too much hydrogen in the film, complicating the proper control of H.^11,16,17^ To solve this problem, an Al_2_O_3_ layer can be used due to its excellent diffusion barrier property against hydrogen.^12,18^ In addition, Al_2_O_3_ is usually deposited through the atomic layer deposition (ALD) method, and the H concentration in Al_2_O_3_ can be controlled by the deposition temperature.^19^

In this work, the effects of H on the characteristics of a-IGZO TFT with Al_2_O_3_ as a gate insulator were examined. The amount of H in the Al_2_O_3_ layer was controlled by varying the deposition temperature (*T*_dep_) used during the ALD method. Additionally, pre-annealing of the Al_2_O_3_ gate insulator was applied to control the H content in the film. The transfer parameters of a-IGZO TFTs such as the hysteresis, sub-threshold swing (S.S.) and mobility were then studied in relation to the H content in the Al_2_O_3_ gate insulator. In addition, according to the post-annealing temperature (*T*_post-ann_), certain changes in the transfer parameters were investigated with regard to H diffusion in an a-IGZO TFT. The results showed that the amount of H in an active channel can easily be changed, and this amount determines the electrical properties of a-IGZO TFTs, including their reliability under bias temperature stress.

## 2. Experiment

**Fig. 1:**
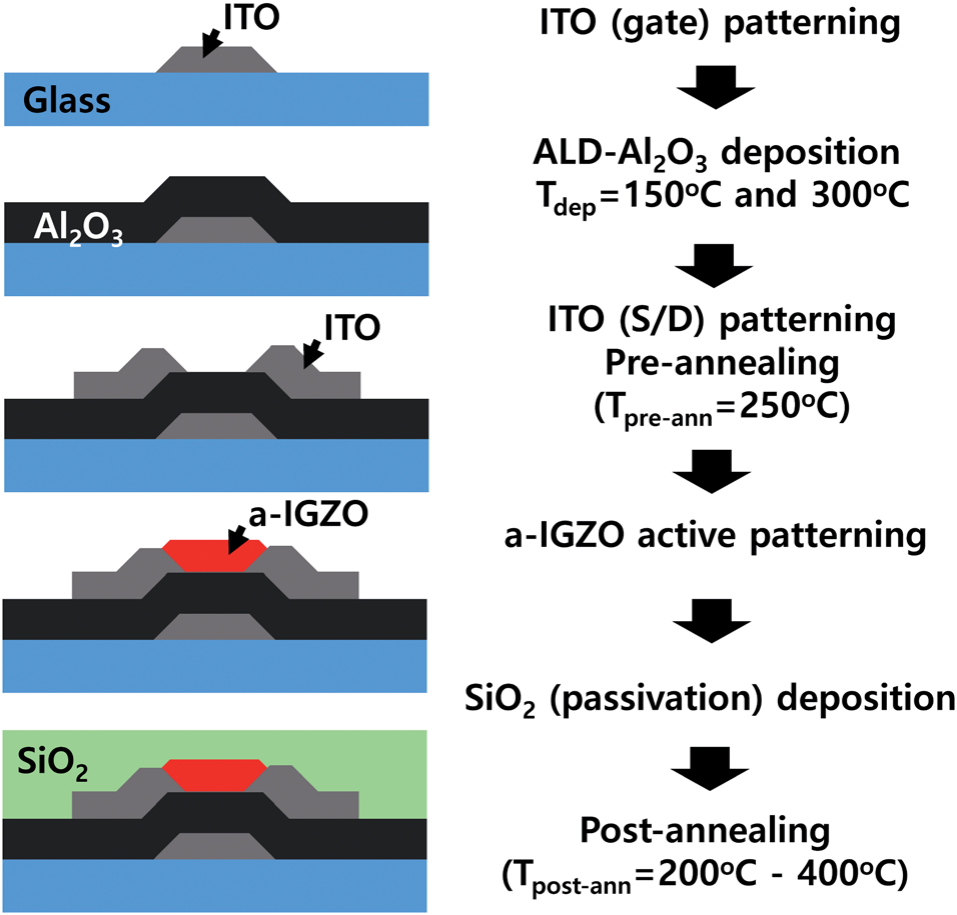
Fabrication procedures for the bottom gate bottom contact (BGBC) a-IGZO TFT with the Al_2_O_3_ gate insulator of *T*_dep_ = 150 °C and 300 °C.

An amorphous indium–gallium–zinc oxide (IGZO) TFT with a bottom gate bottom contact (BGBC) structure was fabricated, the schematic experimental flow of which is shown in Fig. 1. A patterned In–Sn–O (ITO) gate electrode was formed by a wet-etching process using a 150 nm-thick ITO-coated glass substrate. The aluminum oxide (Al_2_O_3_) gate dielectric layers were deposited by atomic layer deposition (ALD) method at deposition temperatures (*T*_dep_) of 150 °C and 300 °C. Tri-methyl-aluminum (TMA, C_3_H_9_Al, 99.9999%) and water were used as an aluminum and oxygen source, respectively. The deposited Al_2_O_3_ layers were wet-etched to open the gate electrode. Then, the 150 nm-thick ITO was deposited by sputtering, followed by wet-etching for the source and drain electrodes. Before the wet-etching of the ITO, the samples were pre-annealed at 250 °C in a vacuum for 2 h to achieve low resistivity and to ensure good etching of the ITO layer. During this step, several samples were additionally annealed at higher temperatures of 300 °C and 350 °C under a vacuum for 2 h to modify the degree of H content in Al_2_O_3_. The active channel of the a-IGZO film (thickness of 40 nm and a metal ratio of In : Ga : Zn = 1 : 1 : 2.5) was deposited by sputtering at room temperature with an Ar/O_2_ gas ratio of 6 : 4 and was then patterned by wet-etching. For a passivation layer, a 100 nm-thick SiO_2_ layer was deposited by plasma-enhanced chemical vapor deposition (PECVD) using silane (SiH_4_) and nitrous oxide (N_2_O) gas at 300 °C. Subsequently, the SiO_2_ layer was etched for the electrode contact. Finally, the fabricated IGZO TFTs were post-annealed in a range of *T*_post-ann_ = 200–400 °C under a vacuum for 2 h.

To determine the amount of hydrogen in the Al_2_O_3_ films, MS-SIMS (IMS 7f, CAMECA) and TOF-SIMS (TOF-SIMS5, ION-TOF GmbH) were used. The cesium (Cs^+^) primary ion beam with current of 15 nA and raster size of 200 μm × 200 μm was used. In addition, the FT-IR microscope (HYPERION 3000, Bruker Optiks) was also used with attenuated total reflectance (ATR) mode. Chemical composition was examined by XPS (K-alpha, Thermo VG Scientific). The data were collected after Ar sputtering for 15 s in an ultra-high vacuum (base pressure of ∼10^−9^ torr). For the calibration, the Ar 2p peak of 241.9 eV was used. The electrical properties of the Al_2_O_3_ gate insulators and a-IGZO TFTs were measured using an Agilent 4284A precision LCR meter and B4156A semiconductor parameter analyser with a probe station.

## 3. Results and discussion

### 3.1. Characteristics of the ALD-Al_2_O_3_ gate insulator deposited at temperatures of 150 °C and 300 °C

**Fig. 2:**
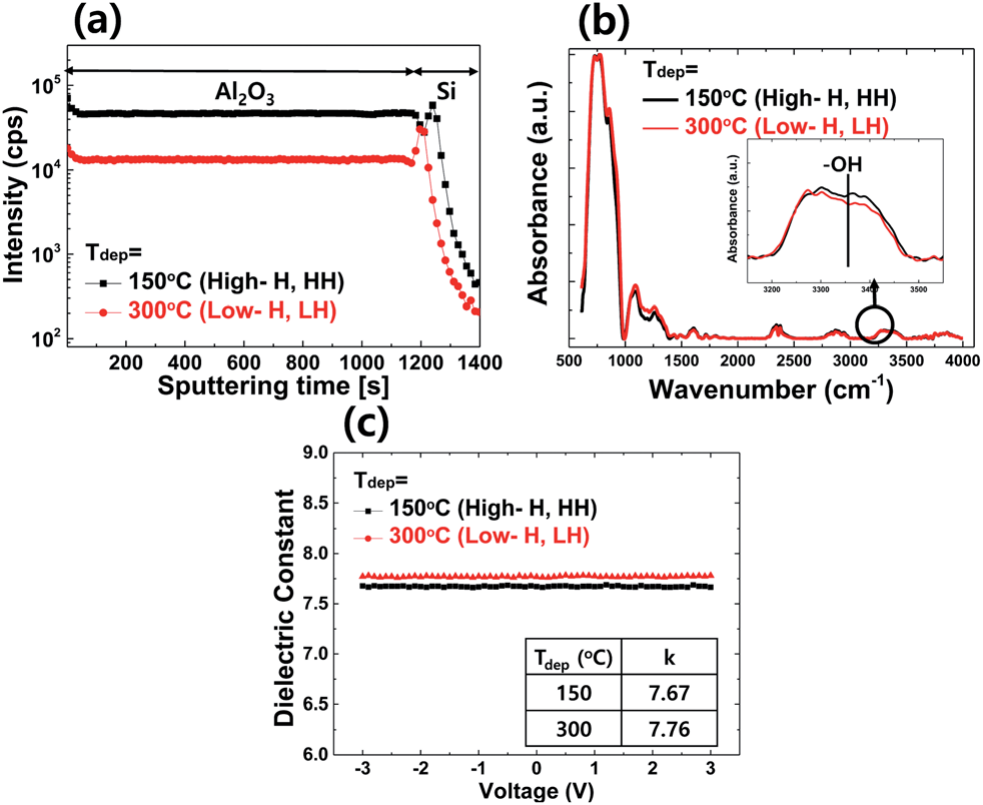
(a) SIMS depth profile results for hydrogen, (b) ATR-FTIR spectra, and (c) dielectric constant (*k*) curves of the ALD-Al_2_O_3_ layer for *T*_dep_ = 150 °C (high hydrogen, HH) and 300 °C (low hydrogen, LH).

First, to reveal the amount of hydrogen of the Al_2_O_3_ depending on *T*_dep_, a SIMS analysis was conducted with the *T*_dep_ = 150 °C and 300 °C films. These results are shown in Fig. 2(a). The SIMS spectra clearly show a difference in the H content on the films; more H with a lower *T*_dep_ for the Al_2_O_3_ film was noted. This result can be easily understood by considering that, during the ALD process, the growth of the Al_2_O_3_ layer is mainly governed by the deposition temperature (*T*_dep_). Generally in ALD, a higher value of *T*_dep_ leads to a lower growth rate and denser film. The residual H in low temperature ALD-Al_2_O_3_ growth occurs as a result of the incomplete removal of the hydroxyl group during the surface reaction. A high *T*_dep_ can facilitate a full sub-reaction between the chemisorbed Al–OH precursor and the gas phase precursor, TMA, by overcoming the steric hindrance of the chemisorbed TMA, resulting in less OH on the surface. From the subsequent reaction between the chemisorbed O–Al(CH_3_)_2_ and water (H_2_O), aluminum hydroxide (Al–OH) is formed, and the formed –OH group further reacts with TMA, resulting in Al–O–Al networks. However, when there are fewer half-surface reactions and an insufficient purge of residual species, hydrogen and/or carbon impurities will remain on the Al_2_O_3_ film. Hence, a high *T*_dep_ readily enhances the surface reaction and the removal of residual species, leading to less H in the Al_2_O_3_ film.^19^ Therefore, the H in the Al_2_O_3_ film is generally considered to be in the form of –OH groups.^20^

Fig. 2(b) shows the ATR-FTIR spectra of the ALD-Al_2_O_3_ film with *T*_dep_ = 150 °C and 300 °C. Strong Al–O stretching vibration was observed in the region of 400–1000 cm^−1^. The broad absorption peak in the range of 3000–3500 cm^−1^ is related to –OH bond stretching vibrations, and this peak was also observed in both Al_2_O_3_ films. This result clearly demonstrates that H mainly exists in the form of hydroxyl groups (–OH) in Al_2_O_3_. In addition, the Al_2_O_3_ film with *T*_dep_ = 150 °C showed a higher –OH peak intensity, in good agreement with the SIMS result (Fig. 2(a)).

The basic characteristics of the ALD-deposited Al_2_O_3_ gate insulator layers, *i.e.* the dielectric constant (*k*) and the chemical composition, including the amount of H, were then examined. Using ITO/Al_2_O_3_/ITO (MIM) devices annealed at a temperature of 350 °C, the dielectric constants (*k*) were extracted. The measured frequency of the capacitance–voltage curves was 1 MHz, and the size of the measured square pad was 300 × 300 μm. The HH- (high hydrogen for *T*_dep_ = 150 °C) and LH- (low hydrogen for *T*_dep_ = 300 °C) Al_2_O_3_ layers showed *k* values of 7.67 and 7.76, respectively, as shown in Fig. 2(c). The higher deposition temperature of the Al_2_O_3_ layer resulted in a slightly increased dielectric constant. As previously reported, the *T*_dep_ of ALD-Al_2_O_3_ scarcely affects the dielectric constant in the range of 150–300 °C.^21^ In addition, after the Al_2_O_3_ layers were annealed up to 400 °C, no changes in the dielectric constant were noted (data not shown). To confirm the chemical composition of the Al_2_O_3_ layers according to *T*_dep_, the XPS analysis was conducted. The XPS results (see Fig. S1†) exhibited only Al 2p and O 1s peaks in all ranges, providing evidence of the formation of an Al_2_O_3_ layer without any impurities, such as carbon-related species. According to the *T*_dep_ value of the Al_2_O_3_ layer, the atomic percentages of Al and O were nearly identical, showing ratios of 42.83 : 57.17 and 43.1 : 56.9 for *T*_dep_ = 150 °C and 300 °C, respectively. In addition, no changes were found after annealing at 350 °C in a vacuum for 2 h.

### 3.2. Changes in the electrical properties of the a-IGZO TFT with ALD-Al_2_O_3_ gate insulator at *T*_dep_ = 150 °C (HH) and 300 °C (LH) depending on the post-annealing temperature (*T*_post-ann_)

**Fig. 3:**
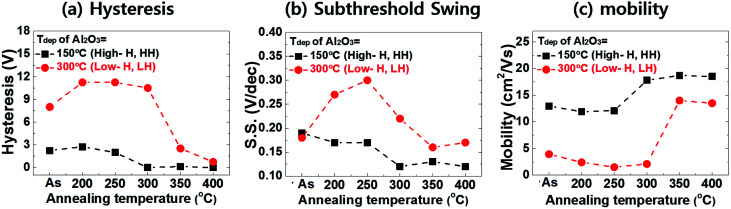
Summary plots of the transfer curve parameters ((a) hysteresis, (b) subthreshold swing and (c) mobility) of the a-IGZO TFTs with the ALD-Al_2_O_3_ gate insulators for *T*_dep_ = 150 °C (high hydrogen, HH) and 300 °C (low hydrogen, LH) according to the post-annealing temperature (*T*_post-ann_ = 200–400 °C).

Fig. 3 shows the variation in the transfer characteristics such as the hysteresis, subthreshold swing (S.S.) and mobility of a-IGZO TFTs with the ALD-Al_2_O_3_ gate insulator for *T*_dep_ = 150 °C (HH) and 300 °C (LH) according to various post-annealing temperatures (*T*_post-ann_ = 200–400 °C). The individual transfer curves and parameters are listed in Fig. S2 and Table S1,† respectively. The transfer parameters of the hysteresis, subthreshold swing (S.S.), and mobility showed large variations and interesting trends according to (1) the hydrogen content in the Al_2_O_3_ gate insulator and (2) the post-annealing temperature of the a-IGZO TFTs.

First, with regard to the hydrogen content, the a-IGZO TFT with the high-hydrogen Al_2_O_3_ gate insulator (HH-device) showed much better transfer properties compared to the low-hydrogen (LH) device throughout the *T*_post-ann_ range of 200–400 °C. In addition, the HH-device exhibited minor variation of the transfer parameters during the post-annealing step. The device showed the best properties at *T*_post-ann_ = 300 °C, becoming nearly saturated when *T*_post-ann_ exceeded 300 °C. In contrast, the LH device showed greatly deteriorated properties, exhibiting much larger hysteresis, S.S. and lower mobility values.

This result can be explained by the different amounts of H in the Al_2_O_3_ gate insulators. As depicted in the SIMS results, the *T*_dep_ = 150 °C Al_2_O_3_ layer has more H in the film compared to the *T*_dep_ = 300 °C sample. This H can easily diffuse toward the a-IGZO active layer during the fabrication and/or post-annealing processes. Here, it should be noted that the SiO_2_ passivation layer was deposited at 300 °C. The H which diffuses from the Al_2_O_3_ gate insulator passivates the defects in the a-IGZO film and improves its TFT properties, as reported in a number of studies.^8–10^ The improvements of the S.S. value and mobility are mainly related to the electron trap sites, which indicates that the devices with higher H concentrations have fewer electron traps in the a-IGZO channel.^9^ In addition, several shallow trap sites located in the gate insulator, generated from the plasma during the a-IGZO deposition step, can also be passivated by H, resulting in an improvement of the hysteresis.^22–24^

Additionally, interesting behaviors of the transfer parameters of a-IGZO TFTs were observed according to post-annealing temperature. First, in the high *T*_post-ann_ (300–400 °C) case, the transfer parameters were dramatically improved. Specifically, this outcome was observed in the LH devices. The LH a-IGZO TFT showed hysteresis of 11.25 V, S.S. of 0.3 V dec^−1^ and mobility of 1.5 cm^2^ V^−1^ s^−1^ at *T*_post-ann_ = 250 °C. These values were improved to 2.52 V, 0.16 V dec^−1^ and 14 cm^2^ V^−1^ s^−1^, respectively, when the device was post-annealed at 350 °C. These parameters were mostly saturated past 350 °C. This large improvement can be attributed to the defect passivation of hydrogen which is diffused to the a-IGZO layer from the Al_2_O_3_ gate insulator during the high temperature annealing process.

**Fig. 4:**
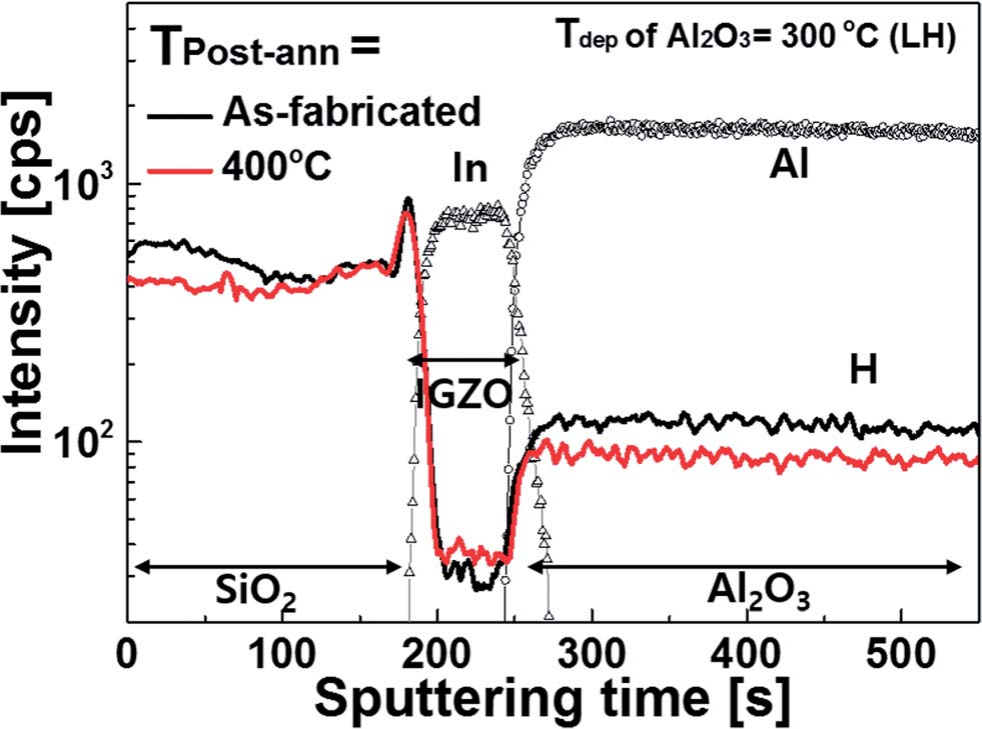
Results of the SIMS depth profile for hydrogen for the SiO_2_/a-IGZO/Al_2_O_3_ structure before and after post-annealing at 400 °C. The Al_2_O_3_ layer is deposited at 300 °C.

The SIMS analysis results (Fig. 4) clearly verify this approach, showing hydrogen diffusion after post-annealing at a high temperature. For the SIMS analysis, a SiO_2_/a-IGZO/Al_2_O_3_ (*T*_dep_ of Al_2_O_3_ = 300 °C) sample, which has a structure identical to that of a TFT, was prepared and annealed at 400 °C. When the annealing at 400 °C, the H intensity in the Al_2_O_3_ layer deceased. On the other hand, in the a-IGZO layer, the amount of H increased. This result strongly suggests that H in the Al_2_O_3_ layer diffuses into the a-IGZO layer, with this causing the passivation of the defects in the a-IGZO layer. Although the Al_2_O_3_ layer is known to be an excellent H diffusion barrier, the effusion of H can take place within several nano-meters of alumina during high temperature annealing at about 400 °C.^25,26^ It is believed that the amount of H which diffused is small because the turn-on voltage (*V*_on_) of the a-IGZO TFT was kept at 0 V during the 400 °C annealing process. Here, it is noted that H in the SiO_2_ passivation layer can also diffuse during the annealing and affect the electrical properties of the a-IGZO TFTs. In the SIMS results, the decreased H intensity in passivation layer (SiO_2_), however, was observed in the surface rather than the inner region of SiO_2_ layer. This indicates that H mainly diffuses toward out to the surface. This could be because the annealing was conducted in a vacuum condition.

On the other hand, in the range of low *T*_post-ann_ (200–250 °C), the a-IGZO TFTs showed more deteriorated transfer curves compared to those of the as-fabricated devices, exhibiting larger hysteresis and lower mobility levels. For the LH device, the hysteresis and S.S. value increased to 11.25 V and 0.3 V dec^−1^ after annealing at 250 °C from 8 V and 0.18 V dec^−1^ (in the as-fabricated case), respectively. The mobility also decreased to 1.5 cm^2^ V^−1^ s^−1^ from 3.95 cm^2^ V^−1^ s^−1^. The results can be explained by the de-passivation effect of H in the a-IGZO layer through the PECVD SiO_2_ passivation film during the post-annealing step. The as-deposited a-IGZO layer contains a large amount of H itself, which is incorporated during the deposition process.^15^ This H can passivate some defect states in the a-IGZO film. However, a heat-treatment could lead to the de-passivation of H, generating new defect states in the a-IGZO layer. Previously, Hanyu *et al.* reported a similar de-passivation effect of hydrogen with dry-O_2_ annealing at *T*_post-ann_ = 400 °C with a-IGZO TFTs without a passivation layer.^9^ In this work, however, it starts at a much lower temperature of about 200 °C; we think that this is attributed to the difference in the device structures and the annealing atmospheres (in this work, a SiO_*x*_ passivation layer and vacuum annealing were used). Actually, Noh *et al.* reported a simulation result which indicates that H_O_ (H occupies the oxygen vacancy site, H_i_ + V_O_) decreases rapidly past an annealing temperature of 180 °C.^10^

Here, it should be noted that the H diffusion effect from the Al_2_O_3_ to the a-IGZO during *T*_post-ann_ = 200–250 °C is minimized due to the pre-annealing process of the Al_2_O_3_ gate insulator at 250 °C. As displayed in Fig. 1, during the fabrication process, there is a pre-annealing step for ITO/Al_2_O_3_/ITO samples at 250 °C in a vacuum for 2 h to improve the ITO quality. During this pre-annealing step, the H in the Al_2_O_3_ layer is effused to a vacuum beforehand, as this step is performed without an a-IGZO layer. Therefore, after the final fabrication of the a-IGZO TFTs, post-annealing below 250 °C can serve only a limited amount of H from the Al_2_O_3_ to the a-IGZO layer. Therefore, for the HH-device shown in Fig. 3, the transfer parameters were greatly improved not at *T*_post-ann_ = 200 °C, but at 300 °C.

**Fig. 5:**
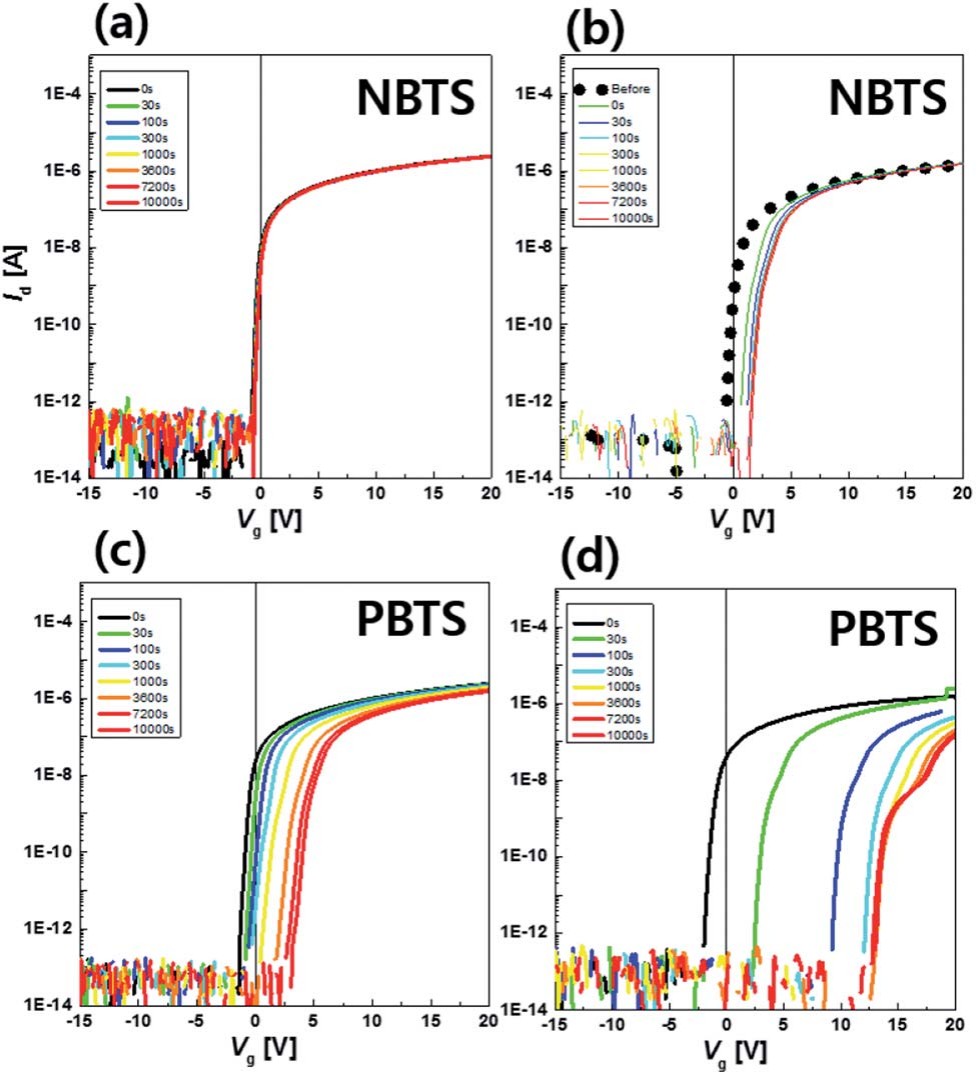
Evolution of the transfer curve of a-IGZO TFTs with Al_2_O_3_ gate insulator for (a) and (c) *T*_dep_ = 150 °C (high hydrogen, HH) and (b) and (d) 300 °C (low hydrogen, LH) under NBTS (*V*_g_ = −20 V, *T* = 60 °C, 10 000 s) and PBTS (*V*_g_ = +20 V, *T* = 60 °C, 10 000 s), respectively. (*T*_post-ann_ = 350 °C).

In addition, the effect of H on the stability was investigated under negative and positive bias temperature stress (NBTS and PBTS, respectively). Gate bias (*V*_g_) levels of −20 V and +20 V were applied at a temperature of 60 °C for 10 000 s for NBTS and PBTS, respectively, using an a-IGZO TFT which was post-annealed at 350 °C. This result is shown in Fig. 5. In the NBTS condition, both devices (HH- and LH-) showed excellent stability, exhibiting *V*_on_ shift values of +0.3 and +1 V, respectively, compared to those of the PBTS condition. This is attributed to the fact that there was a small hole in the a-IGZO film which can be trapped when in the NBTS condition. In contrast, *V*_on_ was greatly shifted by +4.9 and +15.1 V after PBTS for the HH- and LH-devices, respectively. From the parallel shift of the transfer curve without any degradation of the S.S. value, it is suggested that the main reason for the *V*_on_ shift in the PBTS condition is related to the trapping of electrons in the trap sites located in the a-IGZO and/or at a-IGZO/Al_2_O_3_ interface.^27^ During the a-IGZO deposition process, damage at the surface of the gate insulator can be induced by the negative oxygen ion bombardment. This results in a high density of states for electron trapping which becomes more severe upon a higher level of PO_2_.^28^ In this work, a relatively high level of PO_2_ (40%) was used, which would lead to poor stability against PBTS. In addition, the PE-CVD process for the SiO_2_ passivation layer generates excess oxygen in the a-IGZO film, resulting in oxygen interstitial (O–O_i_) related states.^29^ These O_i_ defects easily capture electrons and act as electron traps, resulting in positive shifts of *V*_on_ under positive-bias stress condition.^23,30^ To achieve better stability of the a-IGZO TFTs, an a-IGZO layer with the low PO_2_ condition and/or a protective layer such as ALD-grown Al_2_O_3_ would be introduced.^28^ Though both devices showed poor PBTS stability, it is clear that the HH-device is more stable than the LH-device. This result suggests that the defect passivation effect of H is still valid with regard to PBTS stability for the suppression of electron trap sites.

In addition to the annealing temperature, annealing time can also affect the electrical property and stability of the a-IGZO TFTs. To examine the effect of annealing time, we increased annealing time to 4 h and 6 h for HH- and LH-devices at 350 °C, and results are shown in Fig. S3 and S4.† For the HH-device, the transfer curves showed similar parameters according to annealing time. On the other hand, the LH-device exhibited improved transfer characteristics showing smaller hysteresis and higher mobility as annealing time increased. In the PBTS results, HH- and LH-device showed smaller *V*_on_ shift of +1.54 V and +6.16 V, respectively, after longer annealing process of 6 hours. This results suggest that H continues to diffuse into the active layer from the Al_2_O_3_ layer and passivate defects in the active layer and/or interface between the gate insulator and active.

### 3.3. Changes in the electrical properties of the a-IGZO TFT with an ALD-Al_2_O_3_ gate insulator for *T*_dep_ = 150 °C (HH) depending on the pre-annealing temperature (*T*_pre-ann_)

**Fig. 6:**
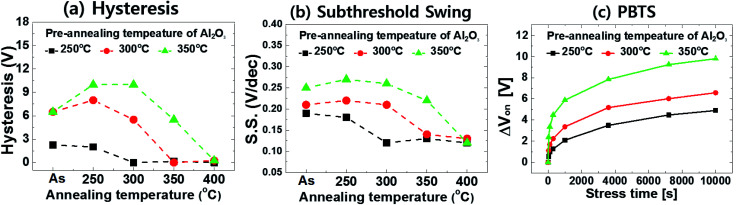
Summary plots of the transfer curve parameters ((a) hysteresis and (b) subthreshold swing) and (c) PBTS stability of the IGZO TFTs according to different pre-annealing temperatures (*T*_pre-ann_) of the *T*_dep_ = 150 °C Al_2_O_3_ gate insulator (HH). The PBTS stability was measured after post-annealing at 400 °C.

As noted above, during the fabrication process of the a-IGZO TFT, there is a pre-annealing step of the Al_2_O_3_ layer at 250 °C which affects the evolution of the transfer parameters according to *T*_post-ann_ due to H effusion. To investigate this approach further, we increased the pre-annealing temperatures to 300 °C and 350 °C from 250 °C in the HH-device. Summary plots of the transfer parameters, in this case the hysteresis and S.S., are shown in Fig. 6(a) and (b), respectively (see Fig. S5, S6 and Table S2 for details†). The result for the HH-device with *T*_post-ann_ = 250 °C is also displayed for comparison as a reference result.

The result clearly showed behaviors identical to those discussed above. First, when *T*_pre-ann_ is increased, the devices showed more deteriorated transfer characteristics in the as-fabricated and low *T*_post-ann_ condition. This results suggest that supply of H from the Al_2_O_3_ layer to the a-IGZO is decreased as *T*_pre-ann_ is higher. During the pre-annealing step of the Al_2_O_3_ layer at the high temperatures of 300 and 350 °C, H is more readily diffused out to a vacuum than 250 °C. Therefore, only a little amount of H can diffuse into the active layer during post-annealing, and it leads to poor transfer curves. In addition, past *T*_post-ann_ = 250 °C, the *T*_pre-ann_ = 300 and 350 °C devices showed worse hysteresis and S.S. values compared to those of the as-fabricated samples. This behavior is identical to that in the LH-devices (see Fig. 3), which have less H in the Al_2_O_3_, and it is related to the de-passivation of H in the a-IGZO layer. This non-passivation effect became more severe as *T*_pre-ann_ was increased because H was more effectively effused and could not be supplied to the active layer. However, when the devices were post-annealed at a higher temperature than *T*_pre-ann_, the transfer characteristics were fully recovered to a level similar to those of the reference devices. The *T*_pre-ann_ = 300 and 350 °C devices showed the recovery of the transfer parameters at *T*_post-ann_ = 350 and 400 °C, respectively. These outcomes indicate that H can be diffused and passivate defects in the a-IGZO layer when annealing takes place at a higher *T*_post-ann_ than *T*_pre-ann_.

The PBTS stability was also measured after *T*_post-ann_ = 400 °C, and the result is shown in Fig. 6(c). Although both devices with *T*_pre-ann_ = 300 and 350 °C showed transfer curves similar to those of the reference sample, the PBTS test showed quite different result. Compared with the Δ*V*_on_ of +4.8 V for the reference, the *T*_pre-ann_ = 300 and 350 °C devices showed large Δ*V*_on_ values of 6.58 and 9.8 V, respectively. These results indicate that, even though defect passivation effect of H can effectively improve the transfer characteristics, there still appears to be other deep traps in the gate insulator.

## 4. Conclusions

In summary, we performed experiments to reveal the effect of hydrogen diffusion on a-IGZO TFTs with Al_2_O_3_ gate insulator. The device with a high level of H in the Al_2_O_3_ exhibited excellent properties, including transfer parameters and bias temperature stabilities as compared to sample with low H levels. The SIMS results showed that H in the Al_2_O_3_ layer was diffused into the a-IGZO layer after post-annealing at 400 °C, suggesting that H has a beneficial effect on the TFT properties in terms of defect passivation. On the other hand, at a low post-annealing temperature (200–250 °C), the devices showed more deteriorated transfer curves compared to those before annealing. This is explained by the effusion of H in the a-IGZO layer through the SiO_2_ passivation layer in terms of de-passivation of H. Additionally, the H contents in the Al_2_O_3_ layer were controlled by varying the pre-annealing temperature, and the defect passivation and de-passivation effects of H were examined in greater depth.

## Conflicts of interest

There are no conflicts to declare.

## Supplementary Material

RA-008-C7RA12841J-s001
