# Supplementary material for: Effect of hydrogen diffusion in an In–Ga–Zn–O thin film transistor with an aluminum oxide gate insulator on its electrical properties
Source: RSC Adv. 2018 Feb 1;8(10):5622–8. doi: 10.1039/c7ra12841j (PMC9078200; doi:10.1039/c7ra12841j)
Supplement: RA-008-C7RA12841J-s001 [file RA-008-C7RA12841J-s001.pdf]

Electronic Supplementary Material (ESI) for RSC Advances.
This journal is © The Royal Society of Chemistry 2018

(Supplementary Information)

Effect of Hydrogen Diffusion in an In-Ga-Zn-O Thin Film Transistor with an Aluminum Oxide Gate Insulator on Its Electrical Properties

Yunyong Nam,[a] Hee-Ok Kim,[b] Sung Haeng Cho,[b] and Sang-Hee Ko Park*[a]

[a] Smart & Soft Materials & Devices Laboratory (SSMD), Department of Materials Science and Engineering, Korea Advanced Institute of Science and Technology (KAIST), 291 Daehak-ro, Yuseong-gu, Daejeon 34141, Korea

[b] ICT Materials & Components Research Laboratory, Electronics and Telecommunications Research Institute (ETRI), 218 Gajeong-ro, Yuseong-gu, Daejeon, 34129, Korea

*Corresponding author: Email address: shkp@kaist.ac.kr

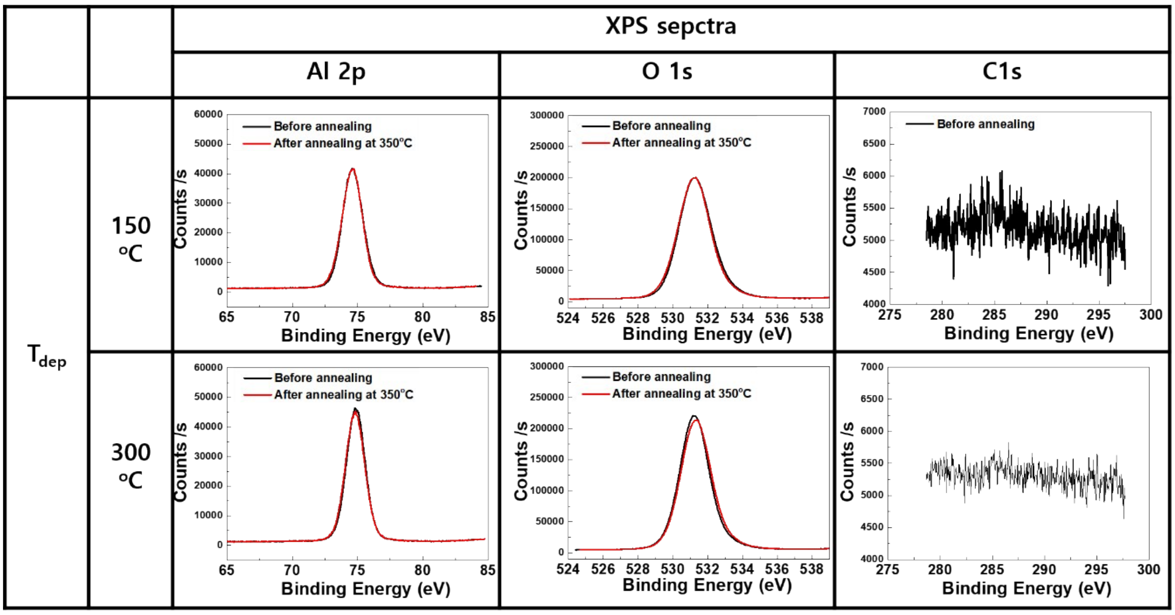

Chemical composition (at%)		
**Depo. Temp.**	**Al**	**O**
150	42.83	57.17
300	43.1	56.9

Fig.S1. XPS spectra (Al 2p, O 1s, and C 1s peaks) of the ALD-Al_2O_3 gate insulators according to deposition temperature before and after post-annealing at 350 ^{o}C in a vacuum for 2 hr. The spectra were callibrated with Ar 2p peak (241.9 eV) after Ar sputtering. The chemical compositions were summarized in the table.

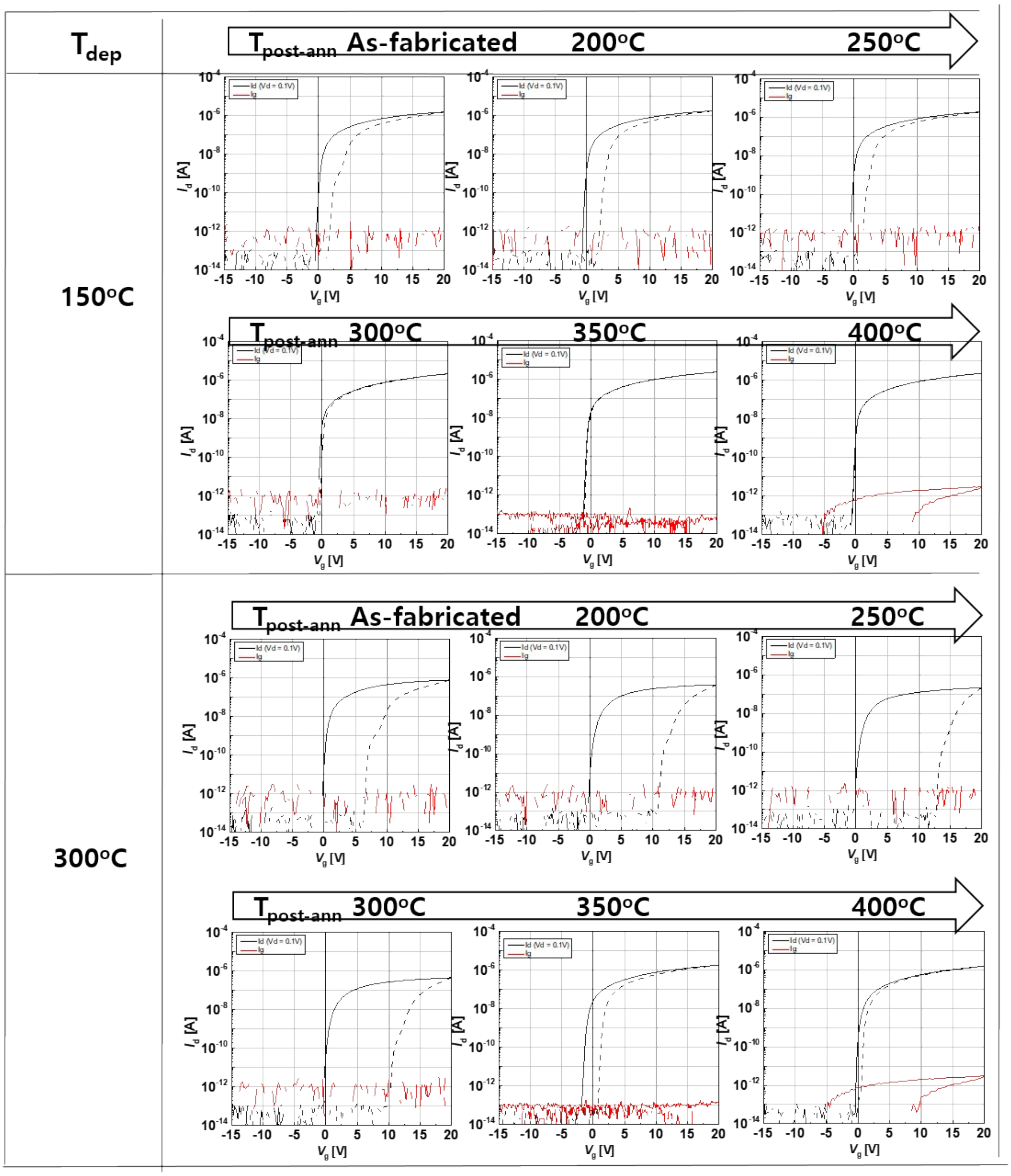

Fig.S2. Individual transfer curves of the a-IGZO TFTs with the Al_2O_3 gate insulators of T_{dep}= 150°C and 300°C according to the post-annealing temperatures ($T_{post-ann}$=200- 400°C)

T_{dep}	Annealing Temp.	S.S	Von	Hys.	u_lin	u_sat
150°C	As	0.19	-0.25	2.25	12.97	10.3
	200°C Vac.	0.18	-0.5	2.75	11.9	10.9
	250°C Vac.	0.18	-0.5	2	12.1	11.76
	300°C Vac.	0.12	-0.75	0	17.8	11.6
	350°C Vac.	0.13	-1	0.14	18.7	12
	400°C Vac.	0.12	-0.5	0	18.5	12.1
300°C	As	0.18	-0.25	8	3.95	7.3
	200°C Vac.	0.27	0	11.25	2.41	4.37
	250°C Vac.	0.3	1.25	11.25	1.5	3.78
	300°C Vac.	0.22	-1	10.5	2.1	4.2
	350°C Vac.	0.16	-1.56	2.52	14	10.1
	400°C Vac.	0.17	-0.25	0.75	13.5	8.7

Table S1. Summary of transfer parameters of the a-IGZO TFTs with the Al_2O_3 gate insulators of T_{dep}= 150°C and 300°C according to the post-annealing temperatures ($T_{post\text{-}ann}$=200- 400°C) (Vds=0.1 V)

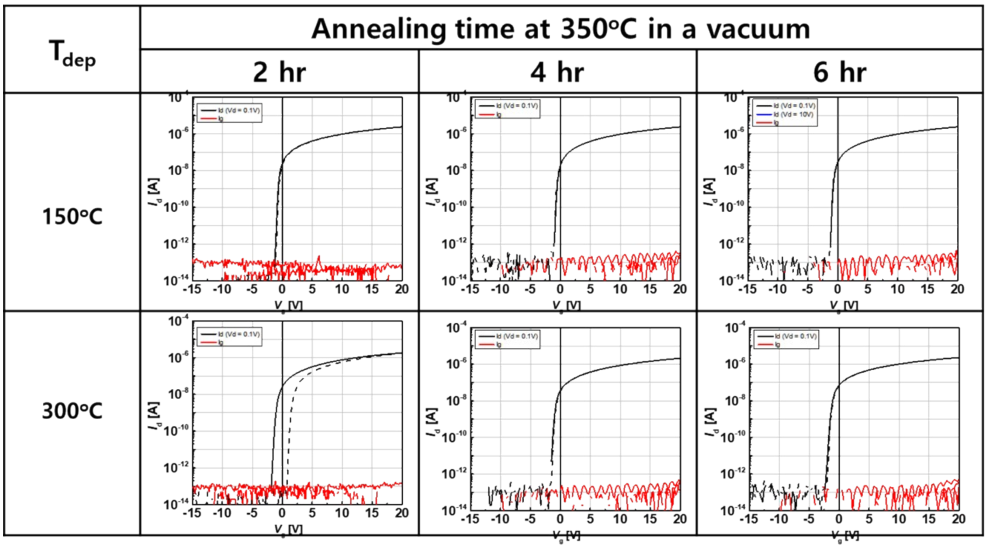

T_{dep}	Annealing Temp.	Time	S.S	Von	Hys.	U_lin	U_sat
150°C	350°C	2hr	0.13	-1	0.14	18.7	12
		4hr	0.13	-1	0	18.8	11.9
		6hr	0.127	-1.25	0	18.84	12.0
300°C		2hr	0.16	-1.56	2.52	14	10.1
		4hr	0.176	-1.5	0.25	16.2	10.3
		6hr	0.18	-2	0.25	16.9	10.8

Fig.S3. Individual transfer curves and summary of parameters of the a-IGZO TFTs with the Al_2O_3 gate insulators of T_{dep}= 150°C (HH-) and 300°C (LH-) according to the post-annealing times (2hr – 6hr). The post-annealing temperature was fixed at 350°C.

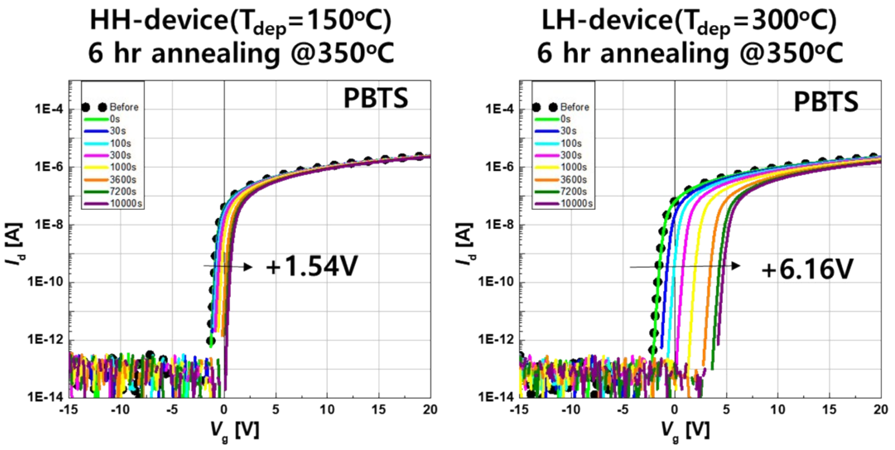

Fig.S4. PBTS stability results of the a-IGZO TFTs with the Al_2O_3 gate insulators of T_{dep}= 150°C (HH-) and 300°C (LH-) after post-annealing for 6 hr at 350°C.

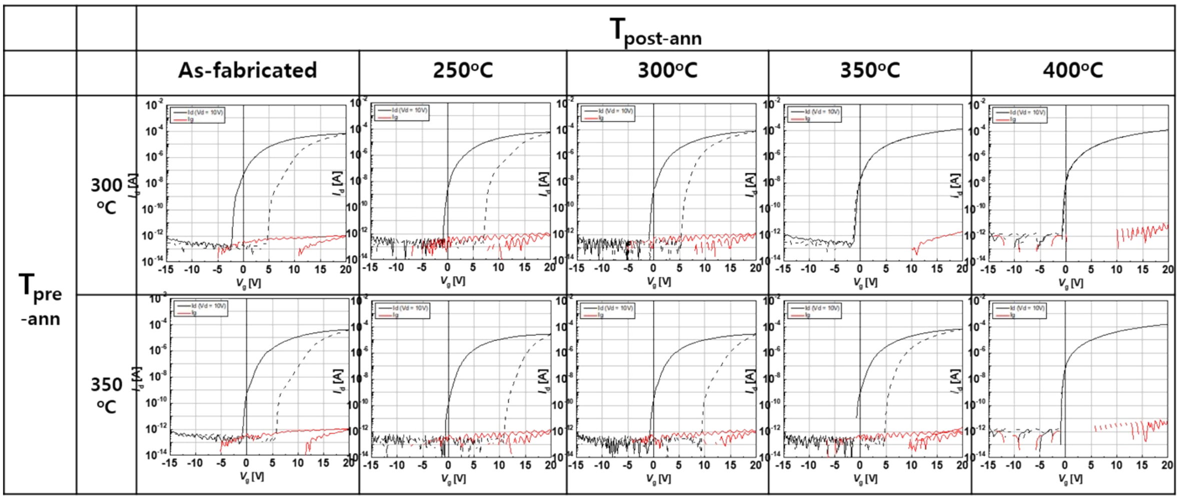

Fig. S5 Individual transfer curves of the a-IGZO TFTs with the Al_2O_3 gate insulators of T_{dep}= 150°C according to different pre-annealing temperatures (300 and 350 °C) of Al_2O_3 layer

Pre-annealing.	Annealing Temp.	S.S	Von	Hys.	Sat_u
300 °C	As	0.21	-1	6.5	5.2
	250°C Vac.	0.22	-0.75	8	5.1
	300°C Vac.	0.21	-0.75	5.5	6.2
	350°C Vac.	0.14	-1	0	7.2
	400°C Vac.	0.13	-1	0.25	10.6
350 °C	As	0.25	-0.5	6.5	3.95
	250°C Vac.	0.27	-0.75	10	2.9
	300°C Vac.	0.26	-0.5	10	2.64
	350°C Vac.	0.22	-0.75	5.5	6.25
	400°C Vac.	0.12	-1.25	0.25	10.3

Table S2. Summary of transfer parameters of the a-IGZO TFTs with the Al_2O_3 gate insulators of T_{dep}= 150°C according to different pre-annealing temperatures (300 and 350 °C) of Al_2O_3 layer

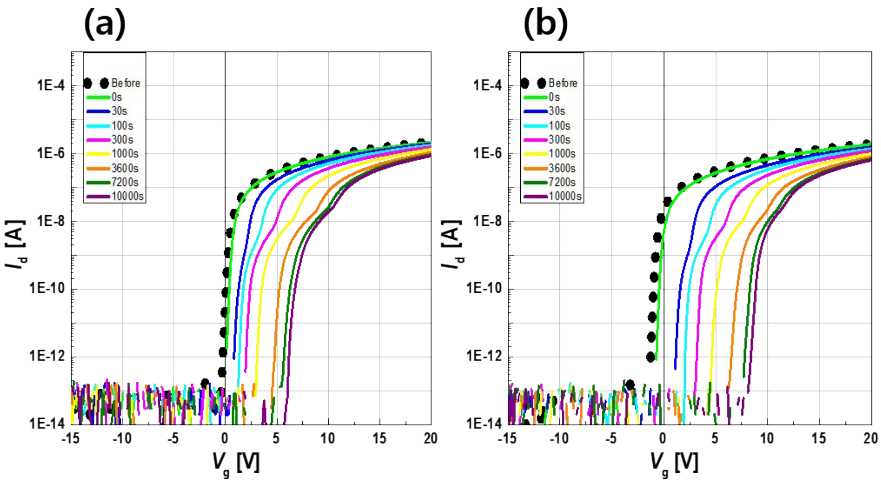

Fig. S6 Evolution of the transfer curve of a-IGZO TFTs with Al_2O_3 gate insulator of T_{dep}=150°C under PBTS condition. (a) $T_{pre-ann}$=300 ^{o}C, and (b) $T_{pre-ann}$=350°C of Al_2O_3 layer. The $T_{post-ann}$ is 400 ^{o}C.
